# Small Bowel Cancer in Crohn’s Disease

**DOI:** 10.3390/cancers16162901

**Published:** 2024-08-21

**Authors:** Ilaria Faggiani, Ferdinando D’Amico, Federica Furfaro, Alessandra Zilli, Tommaso Lorenzo Parigi, Clelia Cicerone, Gionata Fiorino, Laurent Peyrin-Biroulet, Silvio Danese, Mariangela Allocca

**Affiliations:** 1 Department of Gastroenterology and Endoscopy, IRCCS San Raffaele Hospital and Vita-Salute San Raffaele University, 20132 Milan, Italy; faggiani.ilaria@hsr.it (I.F.); damico.ferdinando@hsr.it (F.D.); furfaro.federica@hsr.it (F.F.); zilli.alessandra@hsr.it (A.Z.); parigi.tommaso@hsr.it (T.L.P.); cicerone.clelia@hsr.it (C.C.); sdanese@hotmail.com (S.D.); 2 IBD Unit, Department of Gastroenterology and Digestive Endoscopy, San Camillo-Forlanini Hospital, 00152 Rome, Italy; gionataf@gmail.com; 3 Department of Gastroenterology, Nancy University Hospital, F-54500 Vandœuvre-lès-Nancy, France; l.peyrin-biroulet@chru-nancy.fr; 4 INSERM, NGERE, University of Lorraine, F-54000 Nancy, France; 5 INFINY Institute, Nancy University Hospital, F-54500 Vandœuvre-lès-Nancy, France; 6 FHU-CURE, Nancy University Hospital, F-54500 Vandœuvre-lès-Nancy, France; 7 Groupe Hospitalier Privè Ambroise Parè-Hartmann, Paris IBD Center, F-92200 Neuilly-sur-Seine, France; 8 Division of Gastroenterology and Hepatology, McGill University Health Center, Montreal, QC H4A 3J1, Canada

**Keywords:** small bowel, cancer, Crohn’s disease

## Abstract

**Simple Summary:**

Crohn’s disease (CD) affects the digestive tract, but the ileum is the most frequently involved segment. Compared to the general population, patients with CD are at higher risk of developing small bowel cancer (SBC). However, as it is a very rare event, specific surveillance is not recommended. The pathogenesis of SBC remains unclear, but some risk factors have been identified over time, such as chronic uncontrolled inflammation, long duration of CD, and perianal disease. SBC can manifest with non-specific symptoms, such as abdominal pain, so it can be hard to diagnose. While intestinal ultrasound, magnetic resonance imaging, and video capsule may suggest the presence of SBC, the definitive diagnosis is based on histology. Although there are still very few data on this topic, physicians should be mindful of the risk of SBC in CD.

**Abstract:**

Crohn’s disease (CD) is a chronic inflammatory bowel disease (IBD) that frequently affects the small bowel. Individuals diagnosed with CD are at increased risk of developing bowel cancer compared to the general population. Small bowel cancer is a rare but significant CD complication. Adenocarcinoma represents the most prevalent of these neoplasms, followed by neuroendocrine tumors and sarcomas. The primary risk factors identified are being of the male sex, disease duration, previous surgical intervention, perianal disease, and chronic inflammation. The precise etiology remains unclear. Another crucial issue concerns the role of immunomodulators and advanced therapies. By inhibiting inflammation, these therapies can reduce the risk of cancer, which is often initiated by the inflammation–dysplasia–adenocarcinoma sequence. In accordance with the most recent guidelines, it is not necessary to conduct surveillance in patients with small bowel cancer among CD patients, as it is considered a rare disease. Nevertheless, it is of significant importance for gastroenterologists to be aware of this potential CD complication, as well as the patients who are most at risk of developing it. The purpose of this review is to provide a comprehensive overview of CD-SBC, focusing on epidemiology, etiopathogenesis, risk factors, diagnosis, and the role of advanced therapies in CD-SBC.

## 1. Introduction

Crohn’s disease (CD) is a chronic inflammatory bowel disease (IBD) that may manifest in three distinct phenotypes: inflammatory (non-stricturing and non-penetrating), stricturing, or penetrating [1]. Irrespective of the more or less aggressive nature of the disease, patients with CD are at an increased risk of developing cancers compared to the general population, including small bowel cancer (SBC) [2].

SBC is rare in the general population, with an incidence of less than 5% of gastrointestinal malignancies. In patients with CD, this risk is increased, although it remains relatively low (24.4 per 100,000 person-years) [2]. For this reason, the most recent guidelines on IBD and malignancies do not recommend surveillance for SBC, either by imaging or endoscopy [2]. SBC is characterized by three major histologies: adenocarcinomas, neuroendocrine tumors (NETs), and sarcomas, with small bowel adenocarcinomas (SBAs) representing the most common phenotype (11.2 per 100,000 person-years) [3]. The pathogenesis of SBC in the context of CD is multifactorial, involving chronic inflammation and a pro-carcinogenic microenvironment, caused by the continued activation of the immune response, as indicated by the elevated levels of interleukin (IL)6 and tumor necrosis factor (TNF) and the production of deoxyribonucleic acid (DNA)-damaging reactive oxygen species (ROS) [4,5]. 

The management of patients with CD is complex, particularly in cases of stricturing or penetrating disease and those complicated by the occurrence of cancer in a disease-affected intestinal tract [6]. In this context, SBC introduces a significant and often underappreciated risk. The existing literature on SBC is still limited, with the majority of data derived from retrospective studies. 

The aim of this review is to provide an overview of the epidemiology, etiopathogenesis, risk factors, diagnosis, and the role of advanced therapies in CD-SBC. 

## 2. Materials and Methods

We conducted a comprehensive search of the PubMed, Embase, and Scopus databases up until 30 June 2024, with the aim of identifying studies regarding small bowel cancer in Crohn’s disease. To achieve this, we employed specific search terms such as ‘small bowel adenocarcinomas’, ‘small bowel cancer’, in conjunction with ‘Crohn’s disease’, ‘inflammatory bowel disease’, ‘CD’, ‘IBD’, ‘malignancy’, ‘neoplasia’. We limited our search to articles published in the English language.

Our screening process involved two independent reviewers (IF and FD) who initially assessed titles and abstracts to identify potentially relevant studies. Subsequently, we examined the full texts of these selected articles to determine their eligibility for inclusion. Additionally, we manually scrutinized the reference lists of these articles to ensure that no relevant studies were overlooked during the electronic search. The final inclusion of abstracts and articles was based on their relevance to our research objectives.

## 3. Results

### 3.1. Epidemiology 

Due to the absence of specifically designed population-based studies on this topic, current information regarding the prevalence of SBC in CD patients and their survival rates remains unclear.

According to the latest evidence, a Danish and Swedish population-based cohort study, including 47,370 patients with CD, showed that the incidence rate of SBC is 24.4 per 100,000 person-years, with a 10-year cumulative incidence of 0.29% (95% CI 0.24% to 0.34%). Notably, the incidence of SBC is higher during the first year of follow-up (the adjusted hazard ratio (aHR) was 300; 95% CI 73.4 to 1229) and after 20 years of follow-up (with an increase in the aHR to 2.80; 95% CI 1.64 to 4.78) [4]. A population-based study revealed that patients with CD have a 67-fold increased risk of developing an SBA [7]. A meta-analysis of 53 observational studies including 9642 CD patients showed an almost 30-fold higher risk compared to that in the general population [8]. Another meta-analysis of SBAs in CD patients revealed a prevalence of 1.15 per 1000 CD patients (CI: 0.31–2.33, I^2^ = 85%) [3]. The ileum was the most common site for CD-SBAs, affecting 84% (CI: 0.75–0.91, I^2^ = 71%) of patients. The jejunum and duodenum were less frequently involved, with CD-SBAs affecting these locations at 12% (CI: 0.07–0.20, I^2^ = 66%) and 1% (CI: 0.00–0.04, I^2^ = 0%), respectively [2,3]. After SBAs, NETs were the most common SBC in patients with CD, with an incidence rate of 6.96–9 per 100,000 person-years and an aHR for incidence of NETs of 5.51 (95% CI 3.83 to 7.92) [4,9]. Sarcomas, on the other hand, were a rarer entity: the aHR was 4.04 (95% CI 1.58 to 10.3), with an incidence of 0.95 per 100,000 person-years in patients with CD, compared to 0.29 per 100,000 person-years in the reference group [4] (Table 1).

### 3.2. Etiopathogenesis, Risk Factors, and Molecular Characterization

The etiopathogenesis of SBC in CD is still unclear; however, some modifiable and non-modifiable risk factors, inflammation, and some genetic factors may contribute to its increased incidence in CD patients [4]. CD duration is an important risk factor, tripling the chance of the patient developing SBC over time: 0.4/1000 person-years (95% CI, 0.1/1000–0.8/1000), 0.8/1000 person-years (95% CI, 0.7/1000–1.0/1000), and 1.2/1000 person-years (95% CI, 1.0/1000–1.5/1000) with a disease duration of ≤10 years, >10–20 years, and >20 years, respectively [10,12]. Conversely, disease extension is not considered a risk factor [10]. Abdominal surgery for IBD was found to be a risk factor for developing any type of cancer (OR 1.60, 95% CI 1.23–2.08); as well, perforating behavior compared to non-stricturing, non-perforating behavior was considered a risk factor for any cancer (OR 2.33, 95% CI 1.33–4.11) [13]. Overall, both male and female patients with IBD exhibited a similarly elevated risk of gastric, small bowel, and colorectal cancer (male: OR 1.72, 95% CI 1.32–2.11; female: OR 1.83, 95% CI 1.21–2.45). However, when analyzed in subgroups, males, rather than females, were found to have a significantly higher risk of SBC (male: OR 3.23, 95% CI 1.64–4.83; female: OR 6.87; 95% CI 0.03–13.71) [12].

An association has been identified between SBAs and prior or concurrent ileal dysplasia. This suggests that the disease may arise from chronic ileal inflammation through a dysplasia–adenocarcinoma sequence [4]. In a Danish population study, 90% of CD-SBAs developed in bowel segments affected by CD, with 79% demonstrating a clear progression from inflammation to dysplasia to carcinoma [9]. In this evolution, the mutation of the TP53 tumor suppressor gene and the resulting overexpression of the p53 protein plays a pivotal role in the early stages of IBD-associated cancer [14]. The overexpression of the p53 protein has been demonstrated to promote the activation of the NFkB pathway, which in turn activates IL-6. IL-6 originates from immune cells in the lamina propria and can promote epithelial cell proliferation while also protecting them from apoptosis, paving the way for uncontrolled cell proliferation [13,14]. Interestingly, mutations in isocitrate dehydrogenase 1 (IDH1) and SMAD4 have recently been found to be more common in CD-SBAs compared to sporadic SBAs [15,16]. The SMAD4 mutation was associated with adenocarcinomas exhibiting mucinous morphology, which portends a poorer prognosis, while the most prevalent mutation of IDH1 is the R132C variant, which occurs in 20% of cases [17]. Also, the incidence of p53 overexpression (90% vs. 43%, *p* = 0.012) and methylguanine methyltransferase (MGMT) methylation (75% vs. 22%, *p* = 0.012) was higher in the IDH1 R132C variant associated with CD-SBAs than in sporadic CD-SBAs [16]. In contrast, CD-SBAs have been reported to exhibit a lower incidence of somatic APC and KRAS mutations compared to sporadic SBAs [17].

**Figure 1:**
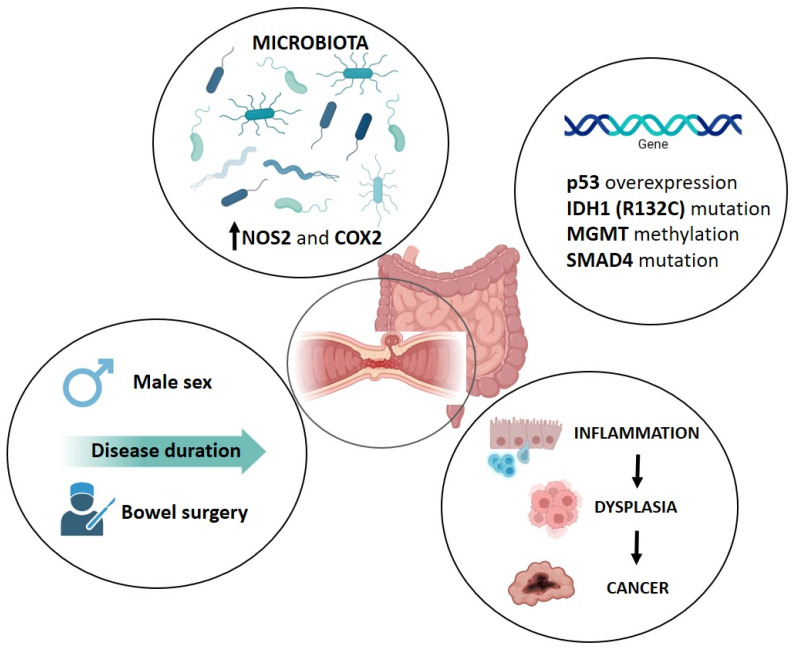
Etiopathogenesis and risk factors of small bowel cancer in Crohn’s disease. NOS2: nitric oxide synthase-2; COX2: cyclooxygenase-2; p53: protein 53; IDH1: isocitrate dehydrogenase 1; MGMT: methylguanine methyltransferase. Arrow (↑): increase.

The role of the microbiota in the pathogenesis of CD and related complications such as cancer is also becoming an increasingly important topic. In fact, the damage to the intestinal barrier can lead to pathological interactions among epithelial cells, microflora, and the immune system. This disruption can result in loss of homeostasis, pathological inflammatory responses, and tumorigenesis [5]. A well-known mechanism concerns the expression of inflammation-related genes such as cyclooxygenase-2 (COX-2) and nitric oxide synthase-2 (NOS-2), which are elevated in inflamed mucosa. In the context of these conditions, the reactive oxygen and nitrogen species produced by the inflammatory cells expressing COX-2 and NOS-2 can accelerate the deterioration of the colonic mucosa and increase its susceptibility to genetic and epigenetic alterations, which in turn can lead to carcinogenesis [14] (Figure 1).

### 3.3. Clinical Presentation and Diagnosis

In patients diagnosed with CD-SBAs, obstruction, weight loss, and abdominal pain are the most common clinical presentations, occurring in approximately 50–68% of cases. In such instances, it is crucial to differentiate between stenosing CD and other potential conditions. This can be challenging, particularly in the absence of specific clinical or laboratory findings [18]. For this reason, CD-SBAs in almost 95% of cases are discovered incidentally during surgical procedures due to stricturing disease [17,19]. 

Calprotectin is a calcium-binding protein whose concentration in feces is directly correlated with the presence of neutrophils in the intestinal lumen. As a result, it is a valuable marker for detecting inflammation associated with IBD in clinical practice [20]. Nevertheless, to date, no studies have evaluated a fecal calprotectin (FC) cutoff that might distinguish between the presence of cancer and the inflammatory reactivation of the disease [21,22]. When a diagnosis is uncertain, it is recommended that endoscopic and imaging examinations be employed to ascertain a clear diagnosis. The diagnostic accuracy of ultrasonography (US), computer tomography (CT), and magnetic resonance (MR) in identifying small bowel CD-associated strictures is high, irrespective of whether the stricture is neoplastic or non-neoplastic [23]. US imaging of luminal diameter <10 mm, wall thickening ≥3–4 mm, and prestenotic dilatation ≥25 mm are suggestive of stricture detection [23]. With regard to the differentiation of CD strictures, no currently available US technique has been demonstrated to have sufficient accuracy to distinguish between inflammation, fibrosis, and underlying cancer [23]. Nevertheless, a retrospective cross-sectional study conducted by Fujita et al., which included 558 consecutive patients, compared the accuracy of intestinal US versus capsule endoscopy and/or balloon-assisted endoscopy to detect SBC [24]. The study yielded that intestinal US had a sensitivity of 53.1% and a specificity of 100%, with a detection rate reaching 91.7% when the SBC was >20 mm [24]. Regarding CT enterography, although not specific, four imaging patterns were associated with SBC: long stenosis with a heterogeneous submucosal layer, short and severe stenosis with proximal small bowel dilation, small bowel mass, and sacculated small bowel loop with irregular and asymmetric circumferential thickening [25]. MR enterography, and in particular (diffusion weighted) DW-MR imaging, is useful for detecting SBAs in patients with CD with a reported sensitivity of up to 100% [26]. Also, following a negative ileocolonoscopy, capsule endoscopy is the most sensitive imaging technique for identifying early mucosal abnormalities in the small intestine. However, in patients with CD, previous cross-sectional imaging or a patency capsule is a mandatory prerequisite for the procedure [27]. In the absence of the expulsion of the patency capsule or the appearance of mucosal changes suggestive of neoplasia on the video capsule, it is imperative to proceed with enteroscopy in order to obtain biopsy specimens [28]. Anterograde or retrograde balloon/spiral-assisted enteroscopy has an 85.9% positive rate of diagnosis [29]. In a single-center Chinese study, 400 patients with or without CD who underwent enteroscopy were evaluated for the presence of small bowel malignancy. Of these patients, 78 (19.5%) were found to have SBC [29]. Moreover, in a cohort study of 101 patients with CD, two patients were found to have indefinite dysplasia based on the results of biopsies taken during an enteroscopy, and one of them was diagnosed with SBA based on the surgical specimen [30]. 

### 3.4. Prognosis 

In patients with CD, the SBA is typically poorly differentiated or undifferentiated, exhibiting signet ring cell histology [16]. This differs from sporadic patients with an SBA, where the tumor is more differentiated. Moreover, tumor specimens from patients with CD who undergo surgical resection are less likely to have positive margins compared to those from patients with sporadic diagnosis (13.6% vs. 19.1%, *p* = 0.01) [31]. Importantly, both higher tumor grade and positive surgical margins are risk factors for decreased survival (HR: 1.09, 95% CI: 1.04–1.14, *p* < 0.001 and HR: 1.60, 95% CI: 1.39–1.84, *p* < 0.001, respectively) [31]. 

Nevertheless, data regarding survival are controversial. Some studies have indicated a numerically higher survival rate for CD-SBA patients compared to sporadic SBA patients (survival rates of 54% vs. 37% at 2 years and 35% vs. 30% at 5 years, *p* > 0.05) [19]. Similar data were obtained using the (American) National Cancer Database, showing comparable overall survival at 5 years between CD-SBA patients and sporadic SBA patients (41% vs. 35%, *p* > 0.05) [31]. This could be due to the fact that CD-SBAs are detected at an earlier stage of disease (I–II) compared to sporadic SBAs (55% vs. 32%, *p* < 0.0001) [32]. A retrospective study by Wieghard et al. including 179 patients with CD-SBAs and 1944 patients with sporadic SBAs revealed that patients with CD-SBAs underwent more surgery than those with sporadic SBAs (81% vs. 72%, *p* = 0.0016), but similar rates of chemotherapy were found (25% vs. 21%, *p* = 0.1886). Overall survival at 5 years was similar between the two groups, except in stage I, where CD-SBA patients demonstrated superior survival compared to sporadic SBA patients (78% vs. 53%, *p* = 0.0576) [32]. Conversely, Axelrad et al. described an increased mortality for CD-SBC: HR 6.26 (95% CI 3.74 to 10.5) [4,33]. The National Comprehensive Cancer Network (NCCN), a group of 33 cancer centers in the United States, has indicated that segmental resection of the small bowel represents the primary course of treatment for SBC. It also has proposed fluoropyrimidine-based adjuvant therapy as a potential option for certain patients, although no studies have yet demonstrated a definitive benefit from this approach [34]. However, according to Fields et al., chemotherapy significantly improved survival in CD-SBA patients (HR: 0.61, 95% CI: 0.53–0.70, *p* < 0.001) [31] (Table 2).

### 3.5. The Role of Immunosuppressants and Advanced Therapies in Crohn’s Disease-Associated Small Bowel Cancer

The use of advanced therapies is a mainstay in the treatment of moderate-to-severe CD. The safety of these drugs has been rigorously tested over time, initially in randomized controlled trials and subsequently in real-world settings [38]. Over the years, there has been evidence that advanced therapies do not elevate the risk of cancer and can be employed with relative safety in patients with a history of or current diagnosis of cancer [2].

Due to the low prevalence of cancer associated with the use of advanced therapeutic modalities (less than 1%), there is a paucity of data pertaining to SBC [39]. A systematic review and meta-analysis conducted by Chin et al. did not identify a significant increase in the risk of CD-SBAs in patients undergoing thiopurine therapy (OR of 1.00; CI: 0.98–1.01, *p* = 0.635) [3]. The use of tumor necrosis factor α (TNFα) inhibitors was not associated with an increased risk of cancer (OR: 0.90; 95% CI: 0.54–1.50) [40]. A multi-center cohort study by Holmer et al., including 125 patients with IBD, yielded that a TNFα antagonist and non-TNF biologics are comparably safe in patients with active or recent cancer. Interestingly, in this cohort, three patients with SBC who had a recurrence of the cancer after being treated with 6-mercaptopurine for CD were included [41]. Regarding other advanced therapies, vedolizumab was not associated with an increased risk of malignancies in a 4-year post-marketing study: 134 (0.35%) out of 37,662 patients with CD were reported having a GI tract tumor, with no differentiation between tumors in the upper bowel, small bowel, and lower GI tract [42]. In this study, the overall risk of malignancies in CD and ulcerative colitis (UC) patients was <1% [42]. Similar data have emerged from ustekinumab use in the UNITI trial. In the three-year follow-up period, which included 576 patients with CD, four cases of tumors occurred, and one of these was an SBA [43]. In the long-term extension of this trial (5 years), the rate of malignancies remained low (1.19 per 100 person-years) [44]. Regarding JAK inhibitors, a meta-analysis by Olivera et al. found that the risk of malignancies was 0.89 per 100 person-years. The most frequent tumor was NMSC (0.51 per 100 person-years), while other tumors were grouped without distinction (0.75 per 100 person-years) [45]. 

## 4. Discussion

SBC represents a rare yet significant complication of CD. Although the incidence is low, patients with CD are at an increased risk of SBC compared to the general population. The risk of developing SBC is elevated in individuals with a longer disease duration, particularly in males, those who have undergone surgical intervention, and those with perianal disease. Furthermore, individuals with involvement of the small intestine, particularly the ileum, are at an elevated risk [4]. 

Moreover, several other factors may contribute to an increased risk of SBC [4]. Chronic inflammation of the intestinal wall represents a substantial risk factor for the promotion of carcinogenesis, via the well-known inflammation–dysplasia–cancer pathway [4]. The alteration of the microbiota that characterizes IBD and the production of proinflammatory molecules such as NOS-2 and COX-2 represent another important factor in terms of genetic, epigenetic, and microenvironment mutations [14]. Ongoing research into biomarkers and genetic factors may improve early detection and personalized treatment approaches. IDH is a gene that encodes a protein involved in the Krebs cycle, specifically the conversion of isocitrate to α-ketoglutarate. The mutation of this gene results in the formation of an oncometabolite, 2-hydroxyglutarate, which alters the Krebs cycle and causes metabolic and epigenetic changes. IDH inhibitors may be a promising therapeutic target in treating CD-SBAs, given that several have been developed and are currently being tested in various clinical trials. For instance, the use of poly(adenosine 5′-diphosphate) ribose polymerase inhibitors (PARPis) has been demonstrated to be beneficial in the treatment of IDH-mutant gliomas and cholangiocarcinomas, due to its capacity to reduce DNA damage [16,46,47]. 

In future studies on SBC, it would be essential to consider how to best manage patients who are at a high risk of cancer. In fact, symptoms of SBC can be non-specific due to overlapping symptoms with CD itself. An elevated FC level may be indicative of underlying inflammation, although it is not a predictor of cancer [22]. Furthermore, FC levels may be influenced by several factors, including the specific test employed, the presence of other gastrointestinal conditions beyond IBD, the use of proton pump inhibitors (PPIs) and nonsteroidal anti-inflammatory drugs (NSAIDs) [22]. The use of US offers a number of advantages, including the absence of radiation, non-invasive procedures, cost-effectiveness, repeatability, and the assessment of the evolution of the disease over a brief period of time [24]. This technique has been demonstrated to have high sensitivity and specificity; however, it is also subject to limitations, particularly with regard to the experience of the operator [24]. The growing application of shear wave elastography (SWE) and strain elastography (SE) represents a significant advancement in the field of ultrasonography [48]. These techniques allow the assessment of fibrotic changes in the CD-affected intestinal tract and, more importantly, enable the differentiation between inflamed and fibrotic tissues based on their stiffness [48]. This is of particular importance in clinical practice and could be the subject of further study in order to establish a possible distinction between benign stenosis and SBC. For instance, the systematic review conducted by Loft et al. demonstrated that endorectal ultrasound, when coupled with elastography, exhibits enhanced sensitivity, specificity, and accuracy in differentiating between rectal adenoma and cancer [49]. Imaging techniques such as CT enterography and MR enterography, are often utilized, but distinguishing between inflammation and neoplastic changes can be complex [25,26,27]. Also, while CRC is more readily identifiable through endoscopic screening and guidelines, as well as the surveillance methods and timelines being more clearly defined, the small bowel presents a more challenging investigation [2,50]. This can be achieved through the use of tests that permit direct visualization of the mucosa of the small intestine, such as capsule endoscopy, preceded by patency in the presence of stenosis, and enteroscopy. The latter technique allows for the biopsy of erosions, ulcers, and stenotic tracts, which might otherwise obscure the presence of dysplasia or cancer [29]. In certain cases, however, a definitive diagnosis of SBC requires the use of laparoscopy and a histological examination of the surgical specimen. Surgery is also necessary in case of intestinal obstruction, perforation, formation of a fistula or abscess, and gastrointestinal bleeding [51]. 

Importantly, there are no specific recommendations for the primary prevention and surveillance of SBC aside from maintaining tight control of inflammation [2]. In the case of a patient exhibiting symptoms of nausea and vomiting following an extended period of asymptomatic status, or in the event that the stricture does not respond to medical therapy, multiplanar imaging is recommended [52]. In accordance with the recommendations outlined in the guidelines for capsule endoscopy, this procedure should be performed in patients who are at elevated risk, including those with long-standing CD (10 years) [28]. The diagnostic yield of capsule endoscopy is enhanced when it is conducted in conjunction with bowel enteroscopy, reaching a rate of 93.3% [53]. Nevertheless, differentiating between an inflammatory stricture and early-stage cancer can often be challenging. Imaging techniques may be unable to detect small lesions and are not always able to differentiate SBC from severe CD [52].

After more than two decades of use, the data regarding the safety of advance therapies are becoming increasingly available. Indeed, the available evidence suggests that they do not significantly increase the incidence of cancer [54]. These data have also been derived from their use in other immune-mediated diseases besides IBD [55,56]. However, it is important to note that patients with a history of malignancy have been excluded from the majority of IBD trials [57], and there are still limited data regarding the safety of using these drugs in patients with a history of cancer or with active cancer [2]. For this reason, data pertaining to the safety in real-world settings are of much significant importance. 

Given the low incidence of CD-SBC and the absence of prospective studies, it is even more challenging to collect meaningful data. Currently, there is limited knowledge about the duration and behavior of CD, the type and duration of medications, disease-free survival, and the detailed molecular phenotypes in patients with CD complicated by SBC. These data are essential for the identification of modifiable risk factors and targets that could be addressed for intervention to prevent the development of CD-SBC. 

## 5. Conclusions

SBC is a known complication of CD. Controlling inflammation through advanced therapies represents the most effective therapeutic weapon to prevent this rare but serious complication. It is essential to conduct population studies, establish registries, perform molecular characterization, and gain a deeper knowledge of the microenvironment in the context of CD. This will facilitate a more comprehensive understanding of the pathogenesis and enable advancements in the prevention, surveillance, and treatment of SBC. 

## Figures and Tables

**Table 1:** The incidence and prevalence of small intestine cancer in Crohn’s disease, as reported in systematic reviews and meta-analyses.

Authors	Publication Year	Study Period	N. of CD Patients	SBC Incidence	SBC Prevalence	Increased Risk of SBC in CD
Laukoetter et al. [10]	2011	1965–2008	40,547	0.3/1000 pyd (95% CI, 0.1–0.5)	0.16% (95% CI, 0.12–0.21)	18.75-fold
Uchino et al. [11]	2020	1955–2019	7344	SIR 22.01 (95% CI, 9.10–53.25)	0.77%	-
Chin et al. [3]	2021	1986–2020	735,136	-	0.11% (95% CI: 0.31–2.33)	-
Wan et al. [12]	2021	2001–2020	531,449 *	-	-	2-fold

* IBD patients. CD: Crohn’s disease; SBC: small bowel cancer; CI: confidence interval; pyd: person years duration; SIR: standardized incidence ratio; N.: number.

**Table 2:** Difference in incidence and prognosis between small bowel cancer, colorectal cancer, and fistula-related cancer in Crohn’s disease.

	Incidence	Prognosis (Mortality)
Small bowel cancer	24.4 per 100,000 person-years [4]	9.00 per 100,000 person-years [4]
Colorectal cancer	0.82 per 1000 person-years [35]	0.47 per 1000 person-years [35]
Fistula-Related cancer	0.2 per 1000 person-years [36]	OS 45.1 ± 28.6 months [37]

OS: overall survival.
